# A Mixed-methods Evaluation of Attitudes and Perceptions of Multispecialty Learners Undertaking a Hybrid Research Methodology Course in Low-resourced Settings

**Published:** 2025-07-11

**Authors:** Surafel Dubale, Marie Martin, Rondi Kauffmann, Jana Macleod, Elizabeth Rose

**Affiliations:** 1 Department of Orthopedic Surgery, AIC – CURE Kijabe Hospital, Kiambu, Kenya; 2 Institute for Global Health, Vanderbilt University Medical Center, Nashville, TN, USA; 3 Department of Health Policy, Vanderbilt University Medical Center, Nashville, TN, USA; 4 Department of Surgery, Kenyatta University, Nairobi, Kenya

**Keywords:** Research training, Research methods, Eastern Africa, Health professions education, Medical research training

## Abstract

**Background:**

Addressing the surgical burden of disease in low- and middle-income countries (LMICs) requires an increased number of medical researchers who understand local systems and resources. Advanced research training in medical programs in LMICs has been limited. To address this need, a hybrid, flipped-classroom research methods course was developed in a nurse anesthetist training program and expanded to surgery and anesthesiology training programs and an anesthesiology faculty development program in two countries. We assessed the effectiveness of the research methods course in strengthening research skills across anesthesiology and surgery specialties by evaluating participants’ attitudes and perceptions of the course.

**Methods:**

Using an online course survey, we collected qualitative and quantitative data on trainees’ perceptions of the course and their self-reported confidence to perform research competencies taught in the course. Quantitative data were analyzed through calculating descriptive statistics, and qualitative findings were reviewed through thematic coding.

**Results:**

Respondents were distributed across the three training programs as follows: surgical residency (n=17), registered nurse anesthetist (n=16), and anesthesiologist faculty development (n=3). Most (94%) agreed that they will use knowledge from this course. Using a scale of 1–5 with 5 corresponding to very confident, the respondents reported being confident in their abilities to engage in all phases of research consistent with the course competencies, with means ranging from 3.83 to 4.11. They were most confident in performing a scientific literature review and identifying a research topic.

**Conclusion:**

This course helped strengthen trainees’ confidence in the seven phases of conducting and disseminating research. The course helped trainees from different specialties and training programs build skills in research methods. This novel, hybrid, flipped-classroom research methods course was effective in building self-confidence for healthcare professionals across the practice continuum to tackle, develop, and perform research.

## Introduction

Rates of death from non-communicable diseases are twice as high in low- and middle-income countries (LMICs) as in high-income countries (HICs) (1). Addressing this burden requires an increased number of LMIC medical researchers who understand local systems and resources (1). Additionally, addressing the deep surgical disparities between LMICs and HICs requires expansion of research activities (2). Further, structured training in research has been shown to increase academic and research productivity in medical fields (3). Strengthening research capacity is a vital approach to improving health systems overall (4).

However, advanced research training in medical programs in LMICs has been limited (5). Some vertical disease-based training programs have included research capacity strengthening within their respective fields [e.g., oral health (6)]. Other programs require individuals to travel to another country for training, such as a 1-day research symposium (7) or a 1-month intensive course (8). Despite being short, program leaders found that symposium participants’ research capacity increased up to 2 years later (7) and course participants submitted 2.6 times more research grants post-course (8). Massive open online courses, such as one on implementation science research by the World Health Organization’s Special Programme for Research and Training in Tropical Diseases, have been used to reach larger audiences and strengthen research skills (9). There remains a continued need to increase locally driven research and training programs and to increase longitudinal programs that are embedded in the local training programs (1).

Responding to needs for increased training in research methods, an embedded research course entitled “Research Methodology” was developed, initially for a Kenyan nurse anesthetist training program. The course has since been adapted and implemented into a Kenyan residency program for junior doctors training in surgery and anesthesia, as well as in anesthesiology training programs in Kenya and Ethiopia. This course is taught by local faculty with virtual support from external international faculty. The course resides on a digital platform that utilizes a hybrid approach to learning.

Our research question focused on addressing the gap in locally driven, sustainable research training for healthcare professionals in LMICs where high disease burdens and surgical disparities necessitate increased research capacity. Building from this question, we had two objectives: (i) describe the innovative hybrid format of the training program and (ii) assess the effectiveness of a local program in training nurses, surgeons, and anesthesiologists in research methods during their clinical training through an evaluation of participants’ attitudes and perceptions of the course.

## Materials and Methods

The course is divided into seven units that guide learners through the steps of developing and implementing comprehensive research. Through this course, learners are equipped with the knowledge and skills necessary to design and implement their own research studies of a high quality that can produce results to advance the practice of healthcare in their regions. Through discussions with program coordinators and local faculty, efforts were made to ensure contextual relevancy. Course resources were packaged into digital modules consisting of pre- and post-tests with each module, journal articles, datasets, independent assignments, group activities, and handbooks for learners and facilitators. The course is designed to be delivered in a “blended learning” or hybrid format, in which learners watch introductory videos on foundational concepts and review online content that are later discussed in class. From the onset of the course, each learner is expected to identify a research question and work concurrently with the course as it teaches the steps of the scientific method to fully develop a research project that is completed by the end of the course.

The course was initially developed for a Kenyan nurse anesthetist training program but has since been adapted and expanded across various cadres of anesthetist providers and trainees in a variety of surgical specialties in Kenya. Other learners, such as Ethiopian anesthetist faculty in a faculty training program, have used the course as an independent study, selecting relevant digital materials from the modules to watch and review.

To evaluate the trainees’ perspectives on and attitudes toward the course and their potential changes in knowledge, an online survey using REDCap (10,11), a secure web-based platform for data collection and storage, was created. A link to the survey was emailed to all trainees at the end of the course between 2017 and 2022. Trainees who did not complete the course were excluded and all trainees who completed the course were included.

The survey included questions about trainees’ perception of the course, the course content, and their confidence in performing the course competencies. The survey included 5-point Likert scales (e.g., 1=strongly disagree, 5=strongly agree; 1=not confident, 5=very confident) that respondents used to quantify their answers and open-response questions to write additional information. Trainees received weekly reminders to complete the course evaluation. Respondents were assured of confidentiality in their responses.

To analyze the quantitative data from the scales, we calculated means and standard deviations. For analyzing the qualitative data from the open-ended questions, we used an inductive thematic analysis to identify salient, common, and recurring themes.

All data were double-coded to maximize the accuracy of the codes and resultant theme developed from the qualitative data. Through discussion among the research team, any disagreements regarding coding of the themes were resolved. Code frequencies and identified overarching themes that summarized the respondents’ perspectives were manually extracted.

Ethical guidelines were adhered to, ensuring respondent confidentiality and anonymity as well as protection of privacy rights by storing data files in a password-protected folder. Ethical approval was received from the associated institutional review boards in Kenya (KH/ISERC/02718/0078/2023) and the USA (VUMC IRB 212198).

## Results

### Quantitative

Since 2017, this course has been conducted with multiple cohorts of trainees across three programs in Kenya and Ethiopia. Of these cohorts, 36 individuals responded to the survey. The respondents represented individuals from a surgical residency program in Kenya (n=17), a Kenyan registered nurse anesthetist training program (n=16), and an Ethiopian anesthesiology faculty development program (n=3). These programs were of varying size, hence the variation in response numbers.

Most respondents (n=34, 94%) agreed or strongly agreed that the course met their expectations and that they will use knowledge gained from this course in their career (Table 1). All respondents affirmed that the course was well designed and that the learning objectives were clearly stated, and most respondents (n=33, 92%) felt that the course units built upon each other.

The respondents rated their confidence in performing each of the seven steps of the scientific method, which is the foundation for the seven units in this course. Using a scale of 1–5 with 5 corresponding to very confident, the respondents were “confident” in their abilities in all areas, with means ranging from 3.83 to 4.11 (Table 2). The respondents felt most confident (mean 4.11) in performing a scientific literature review and in identifying an issue to research. Writing a research hypothesis, designing a research plan, and communicating results were additional areas of confidence.

The respondents also marked how helpful course materials were in enhancing their learning. Most respondents “strongly agreed” or “agreed” that the reading materials, class activities, and learning platform were the most helpful [32 (89%), 32 (89%), 31 (86%), respectively]. For the course assignments, half of the respondents (53%, n=19) reported using Excel and 36% (n=13) reported using no software. Smaller numbers used SPSS inc. (17%, 6; SPSS Inc., Chicago, IL, USA) and Stata (11%, 4; StataCorp, College Station, TX, USA). On average, the respondents reported watching 21–25 of the 35 chapter videos. Three-quarters of the respondents watched the videos only outside of class, while 25% watched videos both in class and outside of class. Prior to the course, only 67% felt “confident” or “very confident” in taking an online course; that number rose to 100% after the course.

### Qualitative

The respondents were asked to provide answers to four open-ended questions, as listed below:

1. Please list three main skills and/or knowledge you gained from the course.
2. Please list three strengths of the course.
3. Please list three weaknesses of the course.
4. Please list any additional topics you think should be included in the course.

### Skills/knowledge gained

Five salient themes emerged in response to the question about skills and knowledge gained from the course. The respondents identified the following as modules that most contributed to the knowledge base: how to conduct a literature review (n=15), data analysis (n=14), study design (n=8), methods of presentation (n=5), and data collection tools (n=5). Additionally, in other questions, the trainees (n=3) noted they learned new types of teaching methods.

### Strengths of the course

When asked about the strengths of the course, the answers varied widely. The most commonly mentioned themes included the following: quality and accessibility of the course videos (n=12) (e.g., “Favours students who aren’t sponsored because they do not need finances to buy textbooks”), excellent presentation and design of slides (n=4), format of lectures (n=4) (e.g., “Enhances student centered learning”), ability of faculty to simplify concepts (n=4), organization of content by lecturers (n=3), and confidence and expertise of the presenters (n=3).

### Weaknesses of the course

Overall, the course was very well received by participants. We asked respondents to identify the weaknesses of the course, and the following themes were identified: internet and bandwidth issues, including associated costs (n=16) (e.g., “Requires a lot of data subscription to access/expensive”), course was too time consuming (n=6), course was too short (n=3) (“It’s a short course yet research takes a longer time”), and videos did not work properly (n=3).

### Suggestions for course improvement

The respondents recommended that the following topics be included in future courses: advanced statistics (n=3), cost of research (n=2), how to write a manuscript for publication (n=2), and how to utilize a reference manager (n=2). A few respondents (n=3) also commented that the course was too basic and should be expanded with more detail (e.g., “It is a crash course. Should be expanded”).

## Discussion

This study reports on the perceptions of learners enrolled in a novel, hybrid, flipped-classroom research course designed to train healthcare professionals across the practice continuum. Our results show that this course helped to strengthen trainees’ knowledge, skills, and confidence in the seven phases of conducting and disseminating research. The trainees reported intending to use this new knowledge and skills in their careers, indicating that the course was perceived as both relevant and applicable. The trainees also reported that they thought that the course was well designed, with clear learning objectives. Overall, the course was well received, and as an added benefit, the trainees reported learning new teaching methods and ways of engaging with the course content.

In the post-coronavirus disease (COVID) pandemic era, virtual educational interfaces remain an important aspect in global medical education programs (12). This study supports the ongoing use of digital, online, and asynchronous learning, as we showed that this hybrid, flipped-classroom model was viewed favorably by learners, allowed flexibility in completing course content when most convenient for the learner, and achieved the stated learning objectives. Similar to other courses that use online materials followed by in-person classes (13), our results showed that the use of pre-class reading combined with facilitated in-class discussion was an effective model for knowledge acquisition. It is also notable that course feedback was consistent across two different countries within East Africa (Ethiopia and Kenya), with trainees from different specialties and of different cadres (surgical residents and nurse anesthetists), and in different types of training programs. One particularly innovative aspect of this course that lends well to different settings and types of students is the different instruction formats that can be tailored to the students’ learning styles. For example, some students used the course asynchronously, while other students used the course within another course. However, in all settings and with all types of trainees, the course was rated very highly.

Future studies should evaluate long-term retention of knowledge obtained from the course, determine whether learners are able to take the knowledge gained and successfully integrate that into their careers, and assess whether individuals who complete the course then go on to train or mentor others in future research.

Despite the overall high ratings for the course, several areas of weakness emerged. The trainees were least confident in data analysis and creating data collection tools. Data collection and biostatistics are complex topics and likely require in-depth training and longitudinal mentorship for the trainees to feel confident. This is an area of future work in the development of subsequent training platforms. Further, although many trainees responded that the pre-class videos were well done and the instructors clearly conveyed information about how to access the videos, interestingly, the videos were not rated as the most useful learning resource. In-class activities were reported as more helpful, suggesting that in-person, synchronous group learning may be more desirable and that asynchronous, digital, online learning without an in-person component may not be as effective. Many of the trainees reported not watching the videos or watching only a part of the videos. Because the videos are a main component in the teaching of content, we surmise that the in-class discussion filled in the content missed in the videos sufficiently. Explanations offered in the qualitative responses may provide a partial explanation for why videos were not widely watched in their entirety. The respondents reported that watching videos required a significant amount of data and bandwidth, which is expensive in many resource-limited settings. Although videos are less expensive than textbooks, and therefore more feasible for use in medical education in resource-limited settings, local internet availability should also be considered when deploying this course in the future.

This study has several limitations. First, the course was utilized in only two countries in East Africa, so our results may not be generalizable to other countries or other contexts around the globe. Trainees from only two specialties, surgical and anesthesia specialties, were included. The results may not be generalizable to trainees from other, non-surgical specialties. In addition, the course was used in different teaching settings, and the variation in classroom instructors may have impacted learning and the impressions of the course. As no pre-course survey was administered, memory recall bias likely affects our results. Finally, without a pre-test to assess trainees’ knowledge at the beginning of the course, true gains in knowledge cannot be determined. Finally, not all students completed the course evaluation, and hence, other important information may have been missed.

## Conclusion

We found that a hybrid, flipped-classroom research training course helped to strengthen trainees’ knowledge, skills, and confidence in the seven phases of conducting and disseminating research. Research capacity strengthening is an important component for improving surgical capacity in underserved settings. The skill of knowing how to ask and answer research questions applicable to the local community is vital and represents a necessary component for improving healthcare. The trainees reported direct application to their future careers, but internet bandwidth limits should be considered when designing courses that require online or digital learning.

## Figures and Tables

**Table 1.** Respondents’ (n=36) that responded “strongly agree” or “agree” on perception of the course

Course aspects	n (%) agreed
The course was well designed.	36 (100%)
The learning objectives of the course were clearly stated.	36 (100%)
This course met my expectations.	34 (94%)
I will use knowledge gained from this course in my career.	34 (94%)
I was able to apply my knowledge to complete course assignments (e.g., small group discussions, video questions, etc.).	34 (94%)
The course units built conceptually upon each other.	33 (92%)
I can independently conduct a research project as a result of taking this course.	30 (83%)
The final exam effectively measured my understanding of the course material.	27 (75%)

**Table 2.** Respondents’ (n=36) confidence in performing the seven steps of the scientific method

How confident are you with each of the following tasks	Mean	SD
Identify problems or issues in healthcare delivery within the context of my work environment.	4.11	0.87
Perform a scientific literature review related to an identified issue.	4.11	0.81
Write a research hypothesis based on an identified issue.	4.03	0.76
Design a research plan that aligns with a research question or hypothesis.	3.97	0.80
Communicate results and analyses of research findings through accurate, scientifically correct writing, presentation, or other forms.	3.97	0.50
Develop a data collection instrument/tool using best practices and based on a research plan.	3.92	0.86
Analyze and interpret qualitative and quantitative data to develop evidence-based conclusions.	3.83	0.80

Scale: 1=not confident; 5=very confident.

SD, standard deviation.

## References

[1] Malekzadeh A, Michels K, Wolfman C, Strengthening research capacity in LMICs to address the global NCD burden. Glob Health Action. 2020; 13(1): 1846904. 33373280 10.1080/16549716.2020.1846904 PMC7782223

[2] Taro T, Yao C, Ly S, The global surgery partnership: an innovative partnership for education, research, and service. Acad Med. 2016; 91(1): 75–8. 26287915 10.1097/ACM.0000000000000859

[3] Wang R, Lucy A, Cochrun S, Preserving the pipeline of surgeon scientists: the role of a structured research curriculum. J Surg Res. 2023; 290: 101–8. 37230044 10.1016/j.jss.2023.04.007

[4] Goto A, Vinih NQ, Van Nguyen TT, Epidemiology research training in Vietnam: evaluation at the five year mark. Fukushima J Med Sci. 2010; 56(1): 63–70. 21485658 10.5387/fms.56.63

[5] Goto A, Nguyen TN, Nguyen TM, Building postgraduate capacity in medical and public health research in Vietnam: an in-service training model. Public Health. 2005; 119(3): 174–83. 15661126 10.1016/j.puhe.2004.05.005

[6] Seminario AL, DeRouen T, Cholera M, Mitigating global oral health inequalities: research training programs in low- and middle-income countries. Ann Glob Health. 2020; 86(1): 141. 33200072 10.5334/aogh.3134 PMC7646278

[7] Wu HH, Ibrahim J, Conway D, Clinical research course for international orthopaedic surgeons: 2-year outcomes. J Orthop Trauma. 2018; 32(Suppl 7): S35–7. 10.1097/BOT.0000000000001292 30247398

[8] Cassell HM, Rose ES, Moon TD, Strengthening research capacity through an intensive training program for biomedical investigators from low- and middle-income countries: the Vanderbilt Institute for Research Development and Ethics (VIRDE). BMC Med Educ. 2022; 22(1): 97. 35164739 10.1186/s12909-022-03162-8 PMC8842898

[9] Launois P, Maher D, Certain E, Implementation research training for learners in low-and middle-income countries: evaluating behaviour change after participating in a massive open online course. Health Res Policy Syst. 2021; 19: 1–2. 33823859 10.1186/s12961-021-00703-3 PMC8025553

[10] Harris PA, Taylor R, Thielke R, Research electronic data capture (REDCap)—a metadata-driven methodology and workflow process for providing translational research informatics support. J Biomed Inform. 2009; 42(2): 377–81. 18929686 10.1016/j.jbi.2008.08.010 PMC2700030

[11] Harris PA, Taylor R, Minor BL, The REDCap consortium: building an international community of software platform partners. J Biomed Inform. 2019; 95: 103208. 31078660 10.1016/j.jbi.2019.103208 PMC7254481

[12] Kejela E, Tesfaye G, Getachew A, Evaluation of knowledge, attitudes, and practice in an online faculty development course for anesthesia educators in East Africa. J Contin Educ Health Prof. 2023; 43(4): 274–8. 37185663 10.1097/CEH.0000000000000493 PMC13286129

[13] Millimouno TM, Delamou A, Kourouma K, Outcomes of blended learning for capacity strengthening of health professionals in Guinea. BMC Med Educ. 2021; 21(1): 406. 34320967 10.1186/s12909-021-02847-w PMC8317298

